# Dynamic Association with Donor Cell Filopodia and Lipid-Modification Are Essential Features of Wnt8a during Patterning of the Zebrafish Neuroectoderm

**DOI:** 10.1371/journal.pone.0084922

**Published:** 2014-01-10

**Authors:** Marta Luz, Stephanie Spannl-Müller, Günes Özhan, Birgit Kagermeier-Schenk, Muriel Rhinn, Gilbert Weidinger, Michael Brand

**Affiliations:** 1 Center for Regenerative Therapies Dresden (CRTD), Technische Universität Dresden, Dresden, Germany; 2 Biotechnology Center, Technische Universität Dresden, Dresden, Germany; Western University, Canada

## Abstract

**Background:**

Wnt proteins are conserved signaling molecules that regulate pattern formation during animal development. Many Wnt proteins are post-translationally modified by addition of lipid adducts. Wnt8a provides a crucial signal for patterning the anteroposterior axis of the developing neural plate in vertebrates. However, it is not clear how this protein propagates from its source, the blastoderm margin, to the target cells in the prospective neural plate, and how lipid-modifications might influence Wnt8a propagation and activity.

**Results:**

We have dynamically imaged biologically active, fluorescently tagged Wnt8a in living zebrafish embryos. We find that Wnt8a localizes to membrane-associated, punctate structures in live tissue. In Wnt8a expressing cells, these puncta are found on filopodial cellular processes, from where the protein can be released. In addition, Wnt8a is found colocalized with Frizzled receptor-containing clusters on signal receiving cells. Combining *in vitro* and *in vivo* assays, we compare the roles of conserved Wnt8a residues in cell and non-cell-autonomous signaling activity and secretion. Non-signaling Wnt8 variants show these residues can regulate Wnt8a distribution in producing cell membranes and filopodia as well as in the receiving tissue.

**Conclusions:**

Together, our results show that Wnt8a forms dynamic clusters found on filopodial donor cell and on signal receiving cell membranes. Moreover, they demonstrate a differential requirement of conserved residues in Wnt8a protein for distribution in producing cells and receiving tissue and signaling activity during neuroectoderm patterning.

## Introduction

Wnt proteins constitute a family of signaling molecules with fundamental roles in pattern formation during animal development and disease [1], [2]. The signaling cascades initiated in cells receiving Wnt signals have been extensively studied ([3], [4], http://www.stanford.edu/group/nusselab/cgi-bin/wnt/). Upon binding of Wnt proteins to their receptors, distinct signaling pathways can be triggered, of which the most thoroughly studied is the canonical pathway, which regulates the activation of Wnt target genes through the stabilization of β-catenin. Wnt/β-catenin signaling regulates patterning of the neuroectoderm during gastrulation [5], [6] and *Wnt8a*, in particular, has been shown to act as a posteriorizing factor in this process [7], [8], [9].

Early signals in the unsegmented neural plate control the formation of fore-, mid- and hindbrain primordia along the anteroposterior (AP) axis. Thereafter, local signaling centers refine the pattern through the control of neighboring cell fate and thus contribute to maintenance of regional identity in the neural tube [10], [11]. One of the best-characterized local signaling centers is the midbrain-hindbrain boundary (MHB) organizer, which induces and maintains positional cell identities in the mid- and hindbrain (reviewed in [12], [13], [14]). The position of the MHB along the AP axis is defined by the interface between the expression domains of two transcription factors: Otx2 in the prospective fore- and midbrain and Gbx2 (Gbx1 in zebrafish) in the future hindbrain (reviewed in [13], [14]). In zebrafish embryos, *wnt8a*, which is expressed in the blastoderm margin at gastrula stages, is directly required for the onset of *gbx1* expression and for the establishment of the posterior border of the *otx2* expression domain in a non-cell autonomous manner [9], [15]. Positioning of the MHB organizer may occur in response to a Wnt8a gradient, but it is unclear how this molecule propagates from its source, the blastoderm margin, to set up such a graded activity in the receiving tissue, the prospective neural plate.

Wnt proteins are hydrophobic due to posttranslational addition of lipids, which might influence their ability to spread through tissue. Mouse Wnt3a contains a palmitate attached to a conserved N-terminal cysteine and a second lipid moiety (palmitoleic acid) is attached to a conserved serine residue [16], [17]. The role of lipid-modifications has been studied by mutation of the conserved amino-acids in several Wnt ligands [18], [19], [20], [21], [22]. Lipidation of the serine is suggested to be important for Wnt secretion [17], while lipidation of the cysteine seems to be required for Wnt activity [16]. However, recent studies show that mouse Wnt1 and Wnt3a without any lipidic adducts are still secreted, albeit at lower levels [23], [24]. Experiments in Xenopus embryos and mammalian cells show considerable differences in signaling activity for the same Wnt cysteine mutant [24]. *In vivo* data from *Drosophila* suggests that lipid-modification at the serine rather than at the cysteine is critical for Wg signaling [22]. Hence, the role of lipid-modifications in Wnt signaling activity seems to differ depending on the specific ligand and/or also on the cellular context. According to the recently solved crystal structure of Xenopus Wnt8 (XWnt8), a lipid is attached to the conserved serine residue but the conserved cysteine residue is engaged in a disulfide bond and cannot therefore serve as a lipidation site [25], demanding a re-evaluation of previous results regarding mutations in these conserved residues. In particular, the specific role of both cysteine and serine residues in Wnt8a signaling and/or patterning of the vertebrate neuroectoderm remains unclear.

To gain insight into the mechanisms involved in tissue distribution of vertebrate Wnt8a, we analysed the dynamic sub-cellular localization of fluorescently tagged Wnt8a in zebrafish embryos *in vivo*. We find that Wnt8a associates with filopodial cellular processes emanating from Wnt8a-expressing cells. The protein can be released to neighbouring tissue from filopodial tips. The striking membrane-bound localization of Wnt8a led us to analyse the role of post-translational lipid-modifications of Wnt8a using in vitro and *in vivo* assays. Expression of a Wnt8a version mutated in the palmitoylated serine results in a reduction of Wnt8a cell and non-cell-autonomous functions as well as secretion. In addition, we show that the conserved cysteine, previously thought to be palmitoylated, is also required for Wnt8a cell and non-cell-autonomous functions and secretion. Interestingly, a double mutation in both cysteine and serine completely abolishes any Wnt8a signaling capability, results in the failure of Wnt8a to regulate gene expression in the neuroectoderm and to induce nuclear translocation of β-catenin *in vivo*. Moreover, signaling-impaired Wnt8a mutants show important differences in membrane and filopodial localization of the protein as well as distribution in the target tissue. These results support a potential importance of filopodial cellular processes in mediating tissue distribution of Wnt8a and illustrate the importance of analysing the function of Wnt8a lipid-modifications in a relevant physiological context.

## Results

### Wnt8a forms punctate structures that associate with filopodia

**Figure 1:**
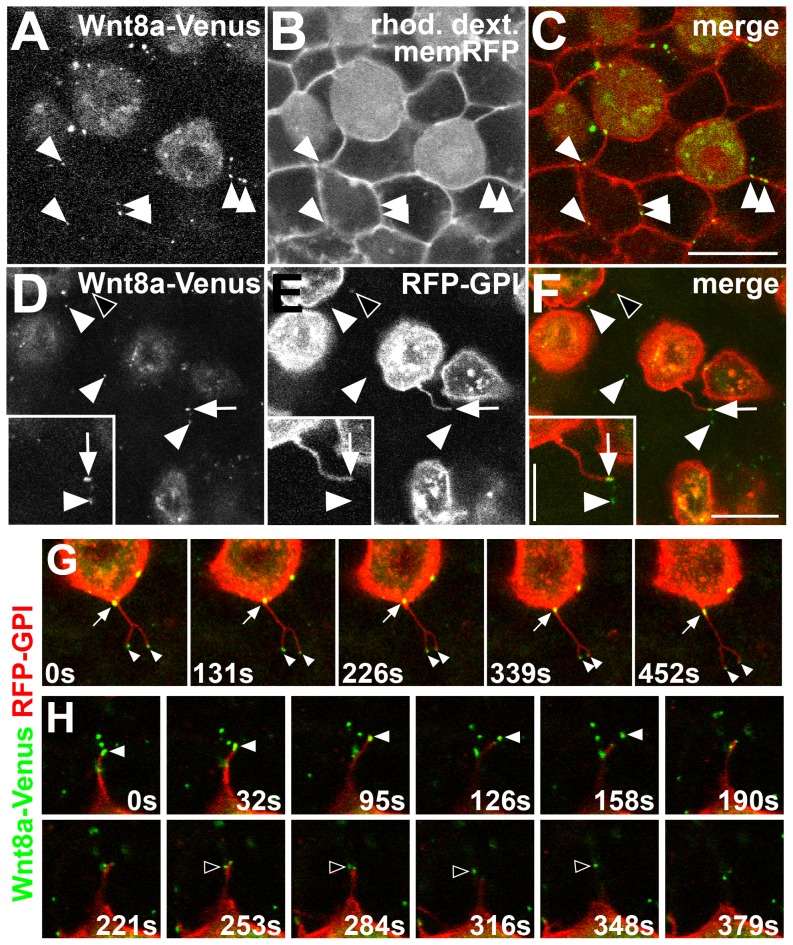
Wnt8a-Venus is membrane associated and released via filopodial processes in the live embryo. Confocal *in vivo* imaging of Wnt8a sub-cellular localization at the animal pole of 60–90% epiboly embryos. (A–C) Wnt8a-Venus expressing cells colabeled with rhodamine dextran (rhod. dextr., cytoplasmic) transplanted into a host embryo expressing membrane-bound RFP (memRFP). (A) Wnt8a-Venus in producing cells and punctate structures in host tissue (arrowheads). (B) Rhod. dextr.-positive, transplanted cells and memRFP-positive cell membranes of the host embryo. (C) Wnt8a-Venus puncta colocalize with the plasma membrane of surrounding host cells (arrowheads). (D–F) Mosaic coexpression of Wnt8a-Venus and RFP-GPI (labeling plasma membrane). Most Wnt8a-Venus puncta do not colocalize with RFP-GPI (arrowheads), some colocalize with filopodia (arrow). RFP-GPI vesicles do not colocalize with Wnt8a-Venus puncta (black arrowhead). Inset shows magnification of filopodia-associated (arrow) and released (arrowhead) Wnt8a puncta. (A,D) Green channel, (B,E) red channel, (C,F) overlay. Scale bars: 20 µm, 10 µm in inset. (G–H) Single frames of confocal time-lapse series on live embryos with mosaic coexpression of Wnt8a-Venus (green) and RFP-GPI (red). (G) A bifurcated filopodia from Wnt8a producing cell containing two Wnt8a puncta at its tips (arrowheads) and a Wnt8a puncta moving from the cell surface into the filopodia (arrow). (H) Two Wnt8a puncta (white and black arrowheads, respectively) are released from a filopodium of a Wnt8a producing cell. Single confocal planes are shown; time is indicated in seconds (s).

During patterning of the neuroectoderm, Wnt8a acts as a posteriorizing factor and positions the MHB, despite being produced by cells located a significant distance away, at the blastoderm margin [9]. The mechanisms of Wnt8a protein release and propagation from its source at the blastoderm margin into the neuroectoderm are not known. To address these mechanims we sought to visualize the protein in live embryos, and tagged Wnt8a at the C-terminus with GFP (Wnt8a-GFP) or the YFP variant, Venus (Wnt8a-Venus). We analyzed the release and distribution of this Wnt8a fusion protein from clones of Wnt8a-Venus expressing cells at the animal pole of wild-type (WT) embryos between 60% and 90% epiboly using *in vivo* confocal imaging. Clones were generated either by mosaic expression or by cell transplantation (see Materials and Methods). To visualize cell membranes of host embryos, we injected mRNA encoding a membrane-bound Red Fluorescent Protein (memRFP). We found that Wnt8a-Venus localizes to punctate structures up to 3 cell diameters away from expressing cells, suggesting that Wnt8a is released from producing cells. The majority of these puncta are found on the plasma membrane of receiving cells (Fig. 1A-C). Similar results were obtained with a Wnt8a-GFP fusion protein [9].

In *Drosophila*, Wg was suggested to propagate in lipoprotein particles, rich in GPI-anchored proteins, called argosomes [26], [27]. To test whether Wnt8a-Venus is released with argosome-like, GPI-rich particles, we co-expressed Wnt8a-Venus with a GPI-linked Red Fluorescent Protein (RFP-GPI) in clones. We observed only very few RFP-GPI-positive particles in the tissue (Fig. 1D-F, black arrowhead). We also observed that the majority of Wnt8a-Venus puncta do not colocalize with RFP-GPI-positive particles in the receiving tissue (Fig. 1D-F, white arrowheads). Importantly, using the same assay, we found that Wnt8a-Venus puncta are present in the cell membrane of Wnt8a-Venus producing cells and that they are often associated with filopodial membrane protrusions (Fig.1 D-F, arrow). These observations suggest that there are two different pools of Wnt8a puncta: one on the membrane and filopodia of the producing cells, and a second, released pool, in the target tissue.

To examine the dynamics of Wnt8a release from producing cells, we followed Wnt8a-Venus puncta in live embryos by time-lapse confocal microscopy. By following individual cells coexpressing Wnt8a-Venus and the membrane label RFP-GPI, we observed that Wnt8a-Venus puncta can translocate from the cell membrane into filopodial protrusions, and can remain associated with filopodia tips for several minutes (Fig. 1G). We also find, though rarely, that Wnt8a-Venus puncta can be released from filopodia of the producing cells (3 puncta, 2/9 embryos, see Movie S1). The low frequency of releasing events recorded is most probably due to the fast nature of the process. An example series of images depicts how two Wnt8a puncta, initially associated with a filopodium, are released as the filopodium retracts towards the cell body (Fig. 1H). This suggests that Wnt8a-Venus may be released from producing cells via filopodia to the signal-receiving tissue.

### Mutations in conserved cysteine and serine affect Wnt8a activity *in vitro*

**Figure 2:**
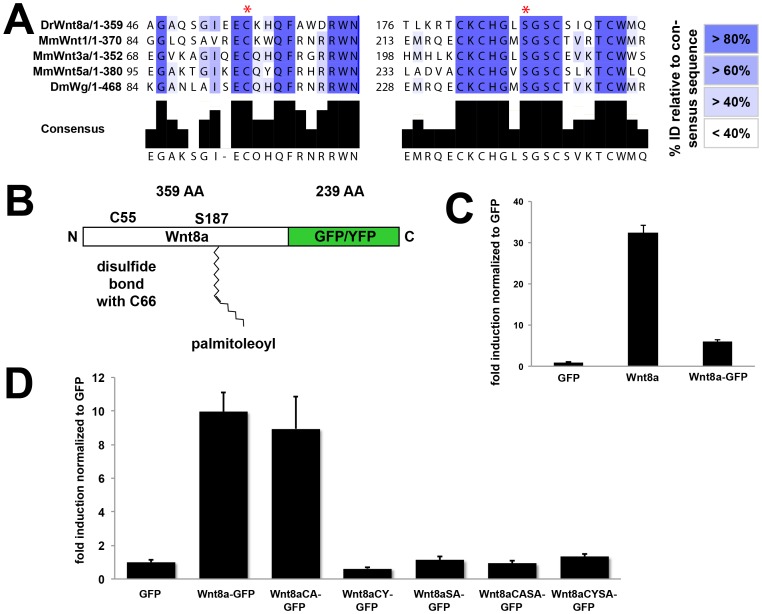
Wnt8a mutant proteins have distinct abilities to activate a Wnt reporter. (A) Amino acid sequence alignments surrounding the conserved aminoacids (red asterisk) for five Wnt proteins. Percentage identity relative to the consensus sequence is colour-coded as described. Dr, *Danio rerio*, Mm, *Mus musculus*, Dm, *Drosophila melanogaster*. (B) Scheme of Wnt8a fusion protein showing conserved residues. (C) pBAR reporter activity (relative to GFP control) in HEK293T cells transfected with untagged and GFP-tagged Wnt8a. (D) pBAR reporter activity (relative to GFP control) in HEK293T cells transfected with the indicated constructs.

We hypothesized that conserved residues of Wnt8a might influence filopodial localization and/or release or distribution of Wnt8a protein. To test the role of the conserved residues in Wnt8a function and tissue distribution, we produced variants of Wnt8a and performed various function tests *in vivo* (Table 1). We identified the conserved residues in zebrafish Wnt8a by sequence comparison to other Wnt proteins, showing that they correspond to a cysteine residue at position 55 and a serine residue at position 187 (Fig. 2A,B). Using the fluorescently tagged Wnt8a, we mutated the cysteine (C) to an alanine (A) or to a tyrosine (Y) generating Wnt8aCA-GFP/Venus and Wnt8aCY-GFP/Venus, respectively. Additionally we mutated the serine (S) to an alanine (A) to generate the Wnt8aSA-GFP/Venus and the same mutation was introduced in the cysteine mutated contructs to obtain the double mutated Wnt8aCASA-GFP/Venus and Wnt8aCYSA-GFP/Venus.

**Table 1:** Summary of activity of different Wnt8a mutants compared to WT Wnt8a-GFP.

	autonomous			non-autonomous	
	Wnt reporter activation	otx2 repression	gbx1 induction	otx2 repression	nuclear beta-catenin
Wnt8a-GFP	++	++	++	++	++
Wnt8aCA-GFP	+	++	+	+	++
Wnt8aSA-GFP	-	+	+	+	++
Wnt8aCASA-GFP	–	–	–	–	–
Wnt8aCY-GFP	–	–	–	–	*
Wnt8aCYSA-GFP	–	–	–	–	ND

++ normal activity; + reduced activity; - no activity; * restricted to 1-cell away from source; ND not determined.

To initially test the activity of the different Wnt8a mutant constructs we used a cell culture assay based on activation of a reporter for TCF/Lef-mediated transcription (pBAR) [28]. In mammalian cells, Wnt8a-GFP showed reduced signaling activity compared to untagged Wnt8a (Fig. 2C). Because antibodies for Wnt8a are not available, tagged Wnt8a is currently the only option to study Wnt8a protein both *in vivo* or in biochemical assays. We therefore compared Wnt8a-GFP mutants to wild-type (WT) Wnt8a-GFP for their ability to induce the Wnt reporter. This assay showed that only Wnt8aCA-GFP was capable of activating the reporter, albeit at lower levels than WT Wnt8a-GFP (Fig. 2D).

### Mutations in cysteine and serine affect Wnt8a activity *in vivo*

**Figure 3:**
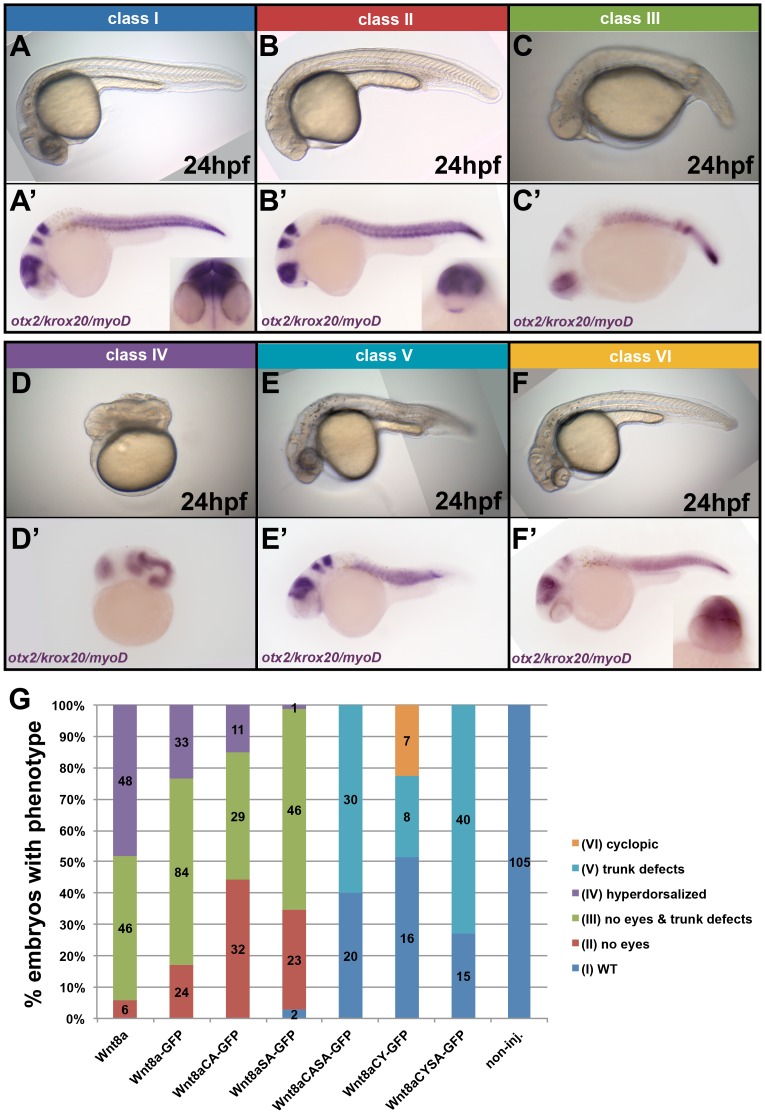
Mutations in Wnt8a affect cell-autonomous neural plate patterning activity. (A–F′) Classes of the phenotypes observed in embryos after overexpression of Wnt8, Wnt8a-GFP or Wnt8a-GFP mutants at 24hpf. (A–F) Live phenotype. (A′–F′) ISH with a probe cocktail including otx2, krox20 and myoD. (G) Distribution of the different phenotype classe upon overexpression of the indicated proteins in percentage. Absolute number of embryos with a given phenotype is shown. Colours correspond to the classes shown in A–F′.

We next compared the biological activity *in vivo* of untagged Wnt8a, WT and mutant Wnt8a-GFP constructs by overexpression in embryos. Wnt8a tagged with either GFP or Venus has identical biological activity as assessed by phenotype at 24hpf after mRNA injection (data not shown), therefore we used both tags interchangeably. Injection of WT and mutant *Wnt8a* mRNAs in one-cell stage zebrafish embryos caused a range of distinct phenotypes. We scored these phenotypes at 24 hpf both in live embryos and after *in situ* hybridization (ISH) using *otx2*, *krox20* and *myoD* as markers and sorted them into six different classes: wt-like (class I), embryos with no eyes (class II), embryos with no eyes and trunk defects (class III), hyperdorsalized embryos (class IV), embryos with trunk defects (class V) and cyclopic embryos (class VI) (Figure 3A–F′).

To compare the activity of untagged Wnt8a, WT and mutant Wnt8a-GFP, we quantified the number of embryos showing the above mentioned phenotypic classes (Fig. 3G). According to the results, wnt8a constructs can be placed in two groups. The first group induces neural posteriorization phenotypes (class II, III and IV) and includes Wnt8a, Wnt8a-GFP, Wnt8aCA-GFP and Wnt8aSA-GFP. Comparing untagged Wnt8a to Wnt8a-GFP, the latter shows an increase in the percentage of embryos with the less severe phenotype (class II) and a decrease in the most severe phenotype (class IV), indicating that Wnt8a-GFP is less active than untagged Wnt8a. The same is observed for Wnt8aCA-GFP and Wnt8aSA-GFP when compared to WT Wnt8a-GFP, indicating that these mutations reduce Wnt neurectoderm posteriorizing activity. The quantifications also suggest that Wnt8aSA-GFP is actually less active than Wnt8aCA-GFP. The second group does not induce neural posteriorization (classesV and VI) and includes Wnt8aCASA-GFP, Wnt8aCY-GFP and Wnt8aCYSA-GFP. Embryos with no eyes (classes II, III and IV) were never observed after injection of Wnt8aCASA-GFP, Wnt8aCY-GFP or Wnt8aCYSA-GFP. Both Wnt8aCASA-GFP and Wnt8aCYSA-GFP injection resulted in embryos only with trunk defects (class V), showing that although they may retain mesoderm patterning activity, they do not affect neurectoderm patterning. Interestingly, overexpression of Wnt8aCY-GFP resulted in a number of embryos showing cyclopia (class VI), a phenotype which is not attributed to Wnt8a overexpression, but normally associated with lack of non-canonical Wnt genes. Surprisingly, this phenotype was not observed upon overexpression of the double-mutant Wnt8aCYSA-GFP.

In summary these results show that: a) Wnt8aCY-GFP protein (in contrast to Wnt8aCA-GFP) is incapable of affecting neuroectoderm posteriorization, suggesting that the mutation of cysteine to tyrosine (instead of alanine) might affect the functional structure of Wnt8a; b) both Wnt8aCA-GFP and Wnt8aSA-GFP show reduced ability to posteriorize the neuroectoderm *in vivo*, compared to WT Wnt8a-GFP, and c) posteriorizing activity is lost in Wnt8aCASA-GFP, which suggests that both residues might cooperate to contribute to a fully functional Wnt8a protein.

**Figure 4:**
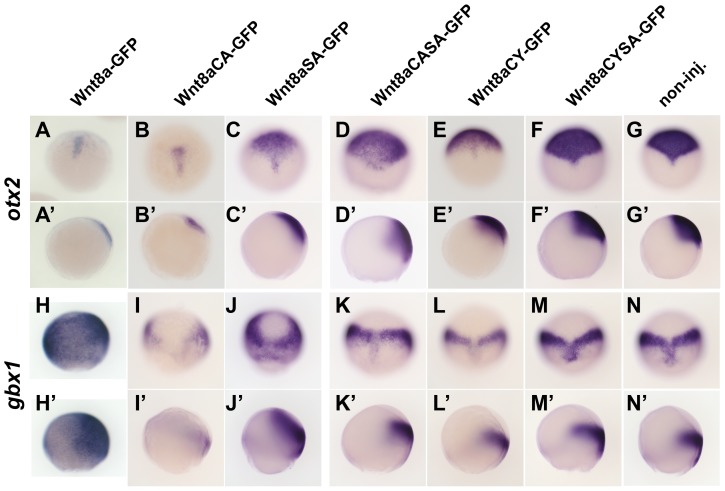
Target gene response to Wnt8a mutant proteins. ISH for *otx2* and *gbx1* after global overexpression of Wnt8a-GFP mutated for lipidation sites compared to Wnt8a-GFP and non-injected control (A,N′) at 80% epiboly. Wnt8a-GFP represses *otx2* (A,A′) and induces *gbx1*(H,H′). Wnt8aCA-GFP is able to repress *otx2* (B,B′) and induce *gbx1*(I,I′). Wnt8aSA-GFP is able to repress *otx* (C,C′) and induce *gbx1*(I,I′). Wnt8aCASA-GFP, WntCYSA-GFP and Wnt8aCY-GFP fail to repress *otx2* (D,E,F, respectively) and induce *gbx1* (J,K,L, respectively). A–F and G–L are dorsal views, anterior to the top; A′–F′ and G′–H′′ are side views, dorsal to the right.

Wnt8a-GFP elicits target gene responses (*otx2* repression and *gbx1* induction) consistent with Wnt8a function in posteriorisation of the neuroectoderm (Fig. 4A-A′;H-H′). To test whether the different Wnt8a mutants also have this ability, we analysed the expression pattern of the *Wnt8a* target genes, *otx2* and *gbx1*, at 80% epiboly (Fig. 4). Expression of Wnt8aCA-GFP leads to repression of *otx2* (Fig. 4B-B′) and *gbx1* (Fig. 4I-I′), which is consistent with high levels of Wnt8a expression [9]. Expression of Wnt8aSA-GFP leads to a weaker repression of *otx2* (Fig. 4C-C′) but induction of *gbx1* (Fig. 4J-J′), which is consistent with low/medium overexpression of Wnt8a [9]. Strikingly, the expression of *otx2* is not repressed by injection of Wnt8aCASA-GFP (Fig. 4D-D′), Wnt8aCY-GFP (Fig. 4E-E′) or Wnt8aCYSA-GFP (Fig. 4F-F′). The expression of *gbx1* is not affected by injection of Wnt8aCASA-GFP (Fig. 4K-K′′), Wnt8aCY-GFP (Fig. 4L-L′) or Wnt8aCYSA-GFP (Fig. 4M-M′). Our data show that only Wnt8aCA and Wnt8aSA are able to affect Wnt8a target gene expression, which results in a posteriorization of the neuroectoderm, consistent with our observations *in vivo*. In summary, these results show that *in vivo*, Wnt8a with a single residue, either cysteine or serine, mutated into alanine, is able to signal, although with reduced efficiency, while mutation of both residues simultaneously renders Wnt8a incapable of signaling and posteriorizing the neuroectoderm. Mutation of the cysteine in the Wnt8a protein to alanine or to tyrosine has different outcomes. While the former results in a protein with reduced signaling ability, the latter results in non-signaling Wnt8a.

**Figure 5:**
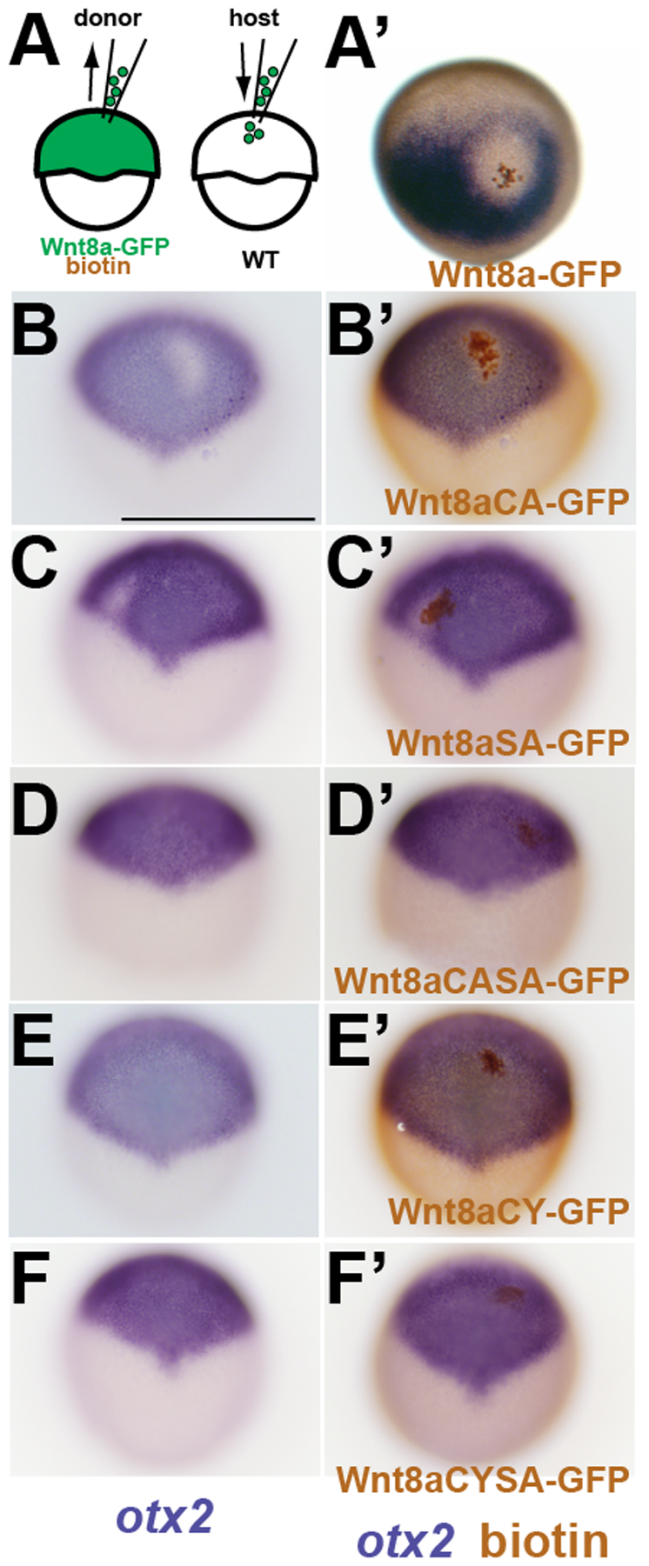
Wnt8a mutations affect cell-non-autonomous neural plate patterning activity. (A) Scheme showing transplantation of cells expressing Wnt8a-GFP or Wnt8a-GFP mutated proteins. (B) Wnt8a-GFP expressing cells (brown) transplanted into a WT embryo at shield stage are able to repress *otx2* (blue) in the surrounding neural plate at 80% epiboly stage (2 hours post transplantation. (A′–F′) Expression of otx2 before (B–F′) and after (A′–F′) detection of transplanted cells. Both Wnt8aCA-GFP and Wnt8aSA-GFP are able to repress *otx2* (B,C) but only cell-autonomously. Wnt8aCASA-GFP, Wnt8aCYSA-GFP and Wnt8aCY-GFP expressing cells fail to repress *otx2* both non- and cell-autonomously. Orientation is anterior to the to the top (D–F).

After testing the signaling activity of mutant Wnt8a proteins upon global expression, we assessed their ability to function non-cell-autonomously. When transplanted into a WT embryo, Wnt8a-GFP expressing cells are able to repress *otx2* in a cell-autonomous but also in a cell-non-autonomous manner up to a distance of 5 cell diameters around the clone (Fig.5A′). This is consistent with our previous findings on the activity of Wnt8a in neuroectoderm patterning [9]. In WT embryos, transplanted cells expressing Wnt8aCA-GFP or Wnt8aSA-GFP are able to repress *otx2* (Fig 5B-C′), but only for 1 to 2 cell diameters around the clones. These single mutations thus appear to abolish long-range functions of Wnt8a. In contrast, transplanted cells expressing Wnt8aCASA-GFP, Wnt8aCY-GFP or Wnt8aCYSA-GFP do not repress *otx2*, not even cell-autonomously (Fig. 5D–F′). Interestingly, Wnt8aSA-GFP expressing cells can repress *otx2* only in the most posterior area of the *otx2* domain, but not anteriorly, suggesting that Wnt8aSA might be more sensitive to Wnt inhibitors which are expressed anteriorly in the neural plate.

### Mutations in conserved cysteine and serine affect Wnt8a-induced non-cell-autonomous β-catenin activation

Upon canonical Wnt signaling, β-catenin accumulates in the cytoplasm and translocates to the nucleus to activate target genes [1]. In WT embryos at shield stage, we detected a nuclear β-catenin staining beyond the Wnt8a expressing domain, at the blastoderm margin, whereas in the animal pole, at this stage, β-catenin staining is restricted to the cell membrane (Figure S1). This indicates active canonical Wnt signaling close to the site of *Wnt8a* expression.

**Figure 6:**
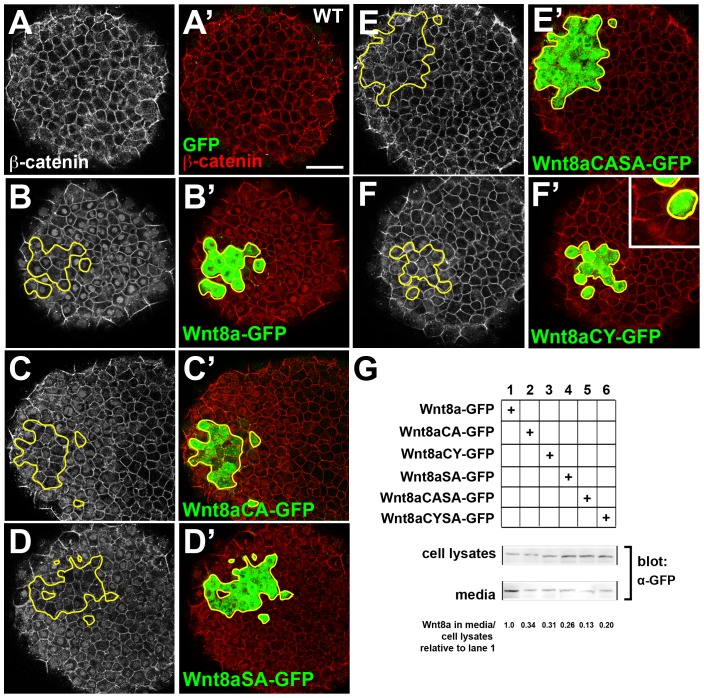
Wnt8a mutations affect cell-non-autonomous activation of β-catenin and secretion. (A–I) Clones of Wnt8a-Venus and Wnt8a-GFP mutants expressing cells transplanted into the animal pole of a WT embryo, fixed one hour after transplantation and stained with anti-GFP (green) and anti β-catenin (red) antibodies. Single confocal sections showing β-catenin is restricted to the membrane in the animal pole of WT embryos (A,A′). Wnt8a-GFP, Wnt8aCA-GFP and WntSA-GFP transplanted cells (B′–D′) induce a wide-range nuclear translocation of β-catenin in the host embryo (B–D) whereas Wnt8aCASA-GFP transplanted cells (E′) do not induce nuclear β-catenin translocation (E). Wnt8aCY-GFP transplanted cells (F′) induce nuclear translocation of β-catenin (F) only in the clone and in host cells directly adjacent to it (inset in F′). Secretion of mutant Wnt8a-GFP proteins is decreased compared to WT Wnt8a-GFP (G). β-catenin in shown in grey (A–F), GFP (green) and β-catenin (red) staining is shown merged (A′–F′). Scale bars: 50 µm.

To determine whether Wnt8a-Venus can induce β-catenin nuclear translocation we transplanted cells from embryos co-injected with Wnt8a-Venus and a lineage tracer into the animal pole of WT embryos, where Wnt8a is not expressed. β-catenin staining was restricted to the cell membrane in the animal pole as seen in WT embryos (Figure 6, S1 and S2). In transplanted embryos fixed 15 minutes after transplantation we could not detect any nuclear β-catenin (Fig. S2D-F). However, a strong nuclear β-catenin staining was observed in the host tissue of embryos one hour after transplantation (Fig. S2G-I). Notably, in the transplanted cells themselves or in embryos injected with Wnt8a-Venus mRNA at the one cell stage nuclear β-catenin staining seemed to be absent or very faint (Fig. S2G-L), which suggest that nuclear β-catenin is down-regulated either upon sustained or excessive Wnt8a signaling. Importantly, these results indicate that Wnt8a-Venus can induce a β-catenin response non-cell autonomously in the living embryo and this tightly time-regulated process can be readily assessed by our transplantation assay.

To determine possible differences between WT Wnt8a and Wnt8a mutants in their capability to induce nuclear translocation of β-catenin, we transplanted cells from embryos injected with fluorescently tagged Wnt8a and Wnt8a mutants into the animal pole of non-injected WT embryos and simultaneously visualized Wnt8a fusion proteins and β-catenin response. Around clones of Wnt8a-GFP expressing cells, fixed one hour after transplantation, we find a strong accumulation of nuclear β-catenin (Fig. 6B,B′; n = 9). Transplantation of cells injected with Wnt8aCA-GFP (n = 8) or Wnt8aSA-GFP (n = 10) lead to the same result (Fig. 6C–D′). In contrast, transplanted cells expressing Wnt8aCASA-GFP did not induce any nuclear accumulation of β-catenin (Fig. 6E,E′; n = 8). Interestingly, cells expressing Wnt8aCY-GFP induce nuclear translocation of β-catenin only in cells directly adjacent to the clone (Fig. 6F,F′, n = 7), even if donor embryos were injected with double or four-fold the amount of Wnt8aCY-GFP (data not shown). In summary, the results of our transplantations show that a) in contrast to the effect on *otx2* repression, Wnt8aCA-GFP and Wnt8aSA-GFP stimulate long-range non-cell-autonomous activation of β-catenin nuclear translocation, b) Wnt8aCY-GFP can stimulate non-cell-autonomous activation of β-catenin nuclear translocation even if only in a paracrine manner and c) the double mutant Wnt8aCASA-GFP does not induce any non-cell-autonomous β-catenin nuclear translocation.

The differences in non-cell-autonomous activity of Wnt8a mutants could be a consequence of altered secretion of Wnt8a proteins. To address whether Wnt8a-GFP mutants are secreted like the WT Wnt8a-GFP, we compared protein levels in conditioned medium and cell lysates (Fig. 6G). We observed a reduction in secretion in all mutants compared to WT Wnt8a-GFP. This reduction could result in the reduced activity observed for Wnt8aCA or Wnt8SA. Secretion of Wnt8aCASA mutant protein seems to be affected most severely, compared to the other mutants, which could in part explain the lack of its signaling activity. Surprisingly, Wnt8aCA or Wnt8aCY show similar levels of secretion, showing that reduced secretion alone cannot account for the differences observed in the signaling activity of these different mutants.

### Non-functional Wnt8a mutations have distinct effects on Wnt8a distribution

**Figure 7:**
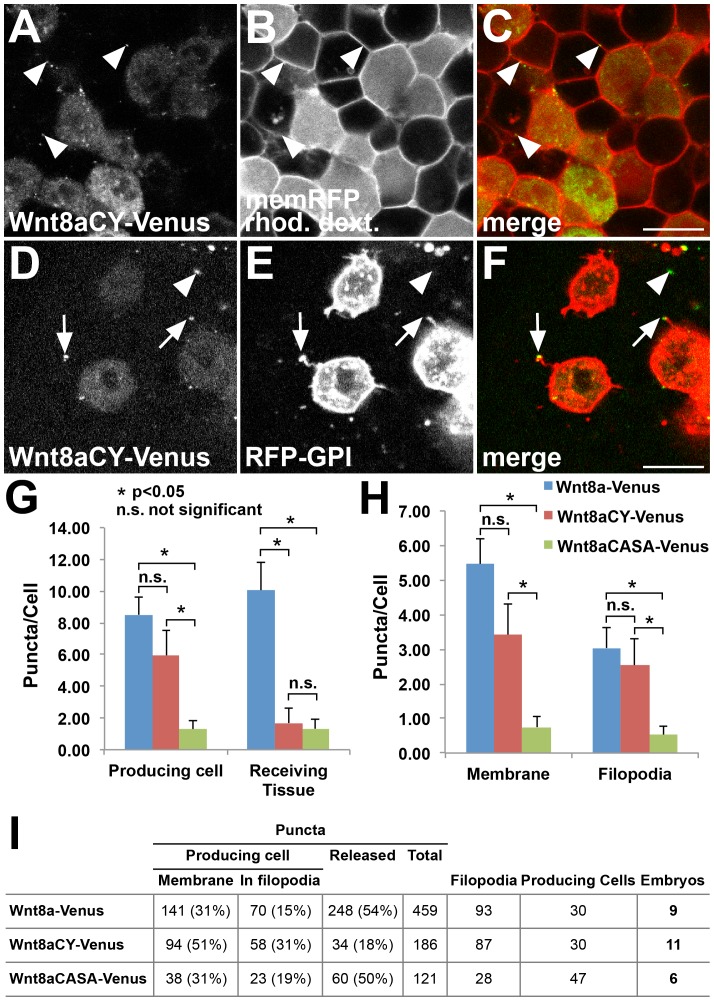
Non-functional Wnt8a mutations have distinct effects on Wnt8a distribution. (A–C) Wnt8aCY-Venus expressing cells colabeled with rhodamine dextran transplanted into a host embryo expressing memRFP. (A) Wnt8aCY-Venus in the expressing cells produce less punctate structures (compare to Fig.1A–C) which nevertheless colocalize with the plasma membrane of receiving cells (arrowheads). (D–F) Mosaic coexpression of Wnt8aCY-Venus and RFP-GPI. The majority of Wnt8aCY-Venus puncta colocalize with filopodia of producing cells (arrow). Only few Wnt8aCY-Venus puncta in the receiving tissue are observed (arrowheads). (G–H) Quantification of the number of puncta in producing cells and in receiving tissue, number of puncta in membrane and in filopodia for Wnt8a-Venus, Wnt8aCY-Venus and Wnt8aCASA-Venus (n = 9 embryos for Wn8-Venus, n = 11 embryos for Wn8-VenusCY, n = 6 embryos for Wnt8aCASA-Venus, error bars represent s.e.m., *p<0.05, n.s. not significant). The number of puncta was normalized to the number of producing cells (labeled with RFG-GPI) in each image. (I) Total numbers for the different parameters analyzed in the quantifications. Producing cells number refers to the sum of RFP-GPI labeled cells in the total number of embryos. (A,D) Green channel, (B,E) red channel, (C,F) overlay. Scale bars: 20 µm. Single confocal planes are shown.

Because the Wnt8aCY and Wnt8aCASA mutants showed the most striking loss-of-function in the previous assays, we decided to analyse them in more detail. We assessed whether the impaired activity of Wnt8aCY-Venus and Wnt8aCASA-Venus correlates with altered protein distribution in the embryo, using confocal *in vivo* analysis. Similar to Wnt8a-Venus, Wnt8aCY-Venus is also found in puncta, which are observed both in producing cells and in the receiving tissue, where they colocalize with cell boundaries (Fig. 7A–C). Co-expression of Wnt8aCY-Venus and RFP-GPI in clones shows that the mutant protein also associates with filopodia of producing cells (Fig. 7D–F). However, the number of puncta in the receiving tissue is strongly decreased (Fig. 7, compare to Fig. 1). We analysed and quantified differences in puncta distribution of Wnt8a-Venus with the signaling defective variants Wnt8aCY-Venus and Wnt8aCASA-Venus. Quantification reveals a significant reduction in the number of puncta in the receiving tissue for Wnt8aCY-Venus while the number of puncta in producing cells is not significantly changed (Fig. 7G). In producing cells, the number of puncta either in the cell membrane or in filopodia is not altered for Wnt8a-Venus compared to Wnt8aCY-Venus (Fig. 7H). In contrast to Wnt8aCY-Venus, Wnt8aCASA-Venus shows reduced number of puncta not only in the receiving tissue but also in the producing cells, both in the membrane as well as in the filopodial fraction (Fig. 7G, H). Thus, while the lack of function of Wnt8aCASA might be attributed to a defective transport to the membrane of producing cells, Wnt8aCY lack of function appears not to be due to defective transport to the membrane and to filopodia but rather from altered distribution in the receiving tissue.

### Wnt8aCY disrupts Wnt-Fzd clusters

**Figure 8:**
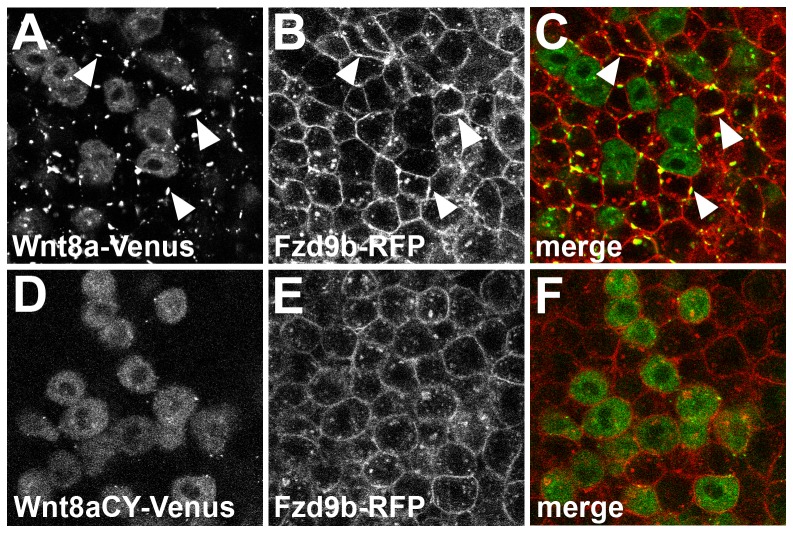
Non-function of Wnt8a results from impaired interaction with its receptor in the target tissue. Single confocal section showing a mosaic expression of Wnt8a-Venus (A–C) or Wnt8aCY-Venus (D–E) in embryos injected with Fzd9b-mRFP mRNA. Co-expression of Wnt8a-Venus and Fzd9b-mRFP induces the formation of membrane clusters which contain both proteins (arrowheads in A–C). Cluster formation is not observed after co-expression of Wnt8aCY-Venus with Fzd9b-mRFP (D-E). (A,D) Green channel, (B,E) red channel, (C,F) overlay.

It has been proposed that canonical Wnt signaling is activated by oligomer formation of Wnt receptors, Frizzled and Lrp [29]. Zebrafish frizzled-5 and frizzled-9b (previously called frizzled-8c and frizzled-9, respectively) have been suggested to be receptors for Wnt8a [30]. To study its distribution *in vivo*, Frizzled9b (Fzd9b) was tagged with RFP. When Fzd9b-RFP was expressed in embryos, we observed that Fzd9b-RFP was normally distributed uniformly at the cell membranes (data not shown). To determine if Wnt8a changes Fzd9b sub-cellular distribution, we expressed Wnt8a-Venus in a mosaic fashion in host embryos that expressed Fzd9b-RFP. We observed the formation of Wnt8a and Fzd9b clusters on the membrane in the host cells (Figure 8A–C, arrowheads). The same experiment was performed using Wnt8aCY. In this case, no clusters of Wnt8aCY-Venus formed with Fzd9b (Figure 8D–E). These results show that Wnt8a and Fzd9b can modulate each others sub-cellular localization, inducing formation of Wnt-Fzd clusters. Moreover, the observed lack of signaling capability of Wnt8aCY correlates with its inability to induce Fzd clustering.

## Discussion

Wnt proteins play key roles in patterning of multicellular animals, acting at a distance from their sites of production. Although their function as morphogens is established, their mode of distribution through target tissue is not clearly understood, and the role of post-translational lipid-modifications affecting Wnt distribution and signaling activity remains elusive. In this study, we report the dynamic association of clustered Wnt8a with filopodial processes of Wnt8a producing cells, suggesting that filopodia-based distribution of Wnt8a may be important for its tissue distribution. We also report non-filopodial clusters of Wnt8a on the surface of receiving cells, that may reflect capturing of Wnt8a into a receptor-ligand complex on receiving cells. To assess a possible role of lipid-modified residues of Wnt8a for signaling and tissue distribution, we studied putative non-lipidated mutant variants of Wnt8a in cultured cells and in distinct *in vivo* assays (Table 1) and discuss here the differences observed as well the implications of our findings for Wnt8a function in patterning of the prospective neuroectoderm.

### Wnt8a localization in the early neural plate in filopodia and in punctate clusters

*Wnt8a in filopodia.* We found that Wnt8a dynamically associates with clusters on filopodia of producing cells, raising intriguing questions about possible functions this might have in the reported long-range signaling activities of Wnt8a. Currently we are not able to selectively disrupt either these filopodia or the localization of Wnt8a to them. However, an involvement of cell protrusions called cytonemes has been suggested to mediate transport of Dpp in the wing imaginal disc of *Drosophila* [31], [32]. While we observe Wnt8a localization on filopodia of Wnt8a *producing* cells, the Dpp-bearing cytonemes emanate from the *receiving* cells. This difference may however not preclude a function in signaling protein propagation, since in cell culture experiments, for example, processes from cells expressing the Shh-receptor Patched can contact filopodia from Shh-expressing cells, resulting in Shh internalization even when using a membrane-bound Shh form [33]. Indeed, a recent study describes specialized filopodia from Shh-producing cells in the developing chick limb bud which together with our observations suggests that filopodial transport of lipid-modified signaling molecules might be a conserved mechanism for intercellular communication [34]. There are also interesting similarities emerging between Wnt8a filopodial localization and Adenomatous polyposis coli (APC) protein localization. APC is a member of the β-catenin destruction complex, forms clusters at cellular protrusions of MDCK cells in a microtubule-dependent process [35] and is required for RNA accumulation in cell protrusions [36]. Recently, Wnt8a was identified as the dorsal determinant in zebrafish, which is displaced to the future dorsal side along functional microtubules [37]. Thus, the relationship between Wnt8a and APC clusters on cell protrusions, and a potential involvement in either APC cytoskeletal functions and/or dorsal determinant localization, will be interesting to study further.

*Wnt8a outside of filopodia.* Outside of producing cells and their filopodia, in the signal-receiving tissue, Wnt8a is mostly localized to punctate clusters of currently unknown nature, which colocalize with Frizzled receptors. These puncta do not colocalize with RFP-GPI labeled vesicles of the producing cells, which argues against a release of Wnt8a through membranous vesicular structures, as suggested eg. for mouse Shh [38]. Absence of RFP-GPI in these Wnt8a puncta also argues against attachment to lipoprotein particles, like the argosomes suggested to transport Wg in the *Drosophila* wing disc [26]. Given the colocalization with Frizzled receptors that we observed, we therefore suggest that the Wnt8a puncta observed in the receiving tissue could be the result of Wnt accumulation in a Frizzled receptor-containing protein signaling complex on signal receiving cells. Similar to Wnt8a, zebrafish Wnt11 induces the formation of patches of its receptor, Frizzled 7, at the plasma membrane in a concentration dependent manner [39]. The formation of these Frizzled7-Wnt11 accumulations depends on the specificity of the ligand-receptor pair, because it is not observed upon combination of Fz7 with another ligand, Wnt3a, or of Wnt11 with an Fgf receptor [39]. We thus suggest that Wnt8a induces similar clustering of Wnt8a ligand-Fzd receptor clusters on signal receiving cells. The crystal structure of XWnt8 and Fzd8-CRD complex suggests the presence of an interaction site mediating the formation of an asymmetric Wnt/Fzd dimer which supports an oligomerization model [25]. Consistent with this possibility, we did not observe the formation of clusters when Fzd9b was combined with the signaling defective Wnt8aCY-Venus mutant (see below); the lack of function of this protein might result from a conformational change that would disrupt the formation of such Wnt/Fzd oligomers.

### Role of conserved residues in Wnt signaling

Several secreted signaling molecules are lipid-modified, but the role of these modifications still remains controversial [40], [41]. While it has been thought that Wnts are modified by the addition of two lipids at a conserved cysteine and a conserved serine, recent evidence indicates that only the serine is indeed modified [25], [42]. Mutations in the conserved serine, as in the Wnt8SA mutant used in our study, should therefore result in a non-lipidated Wnt protein. Consistent with results obtained for Wnt1 and Wnt3a [23], Wnt8aSA is not capable of activating a Wnt reporter in a cell culture signaling assay suggesting that the serine mutation is critical for β-catenin dependent signaling *in vitro*. Surprisingly, Wnt8SA still retains some activity *in vivo* which indicates that in living organisms compensatory mechanisms operate to partially compensate the lack of palmitoylation.

The crystal structures of XWnt8 and DWtnD show that the conserved cysteine previously thought to be lipidated is instead involved in a disulfide bond which should be present in all Wnt molecules considering the high degree of conservation in this residue [25], [42]. Interestingly, at position C55 we observe different mutant Wnt8a protein properties for Wnt8aCA and Wnt8aCY exchanges at the same position. Whereas Wnt8aCA appears to have partially lost protein function, Wnt8aCY protein seems to have lost its function more completely, similar to the Wnt8aCASA mutant. A mutation in Drosophila *wingless* converting the conserved cysteine (C) into a tyrosine (Y) similarly results in a *wingless* loss-of-function phenotype [43] with Wingless protein not being secreted [44], and lacking signaling activity in cultured cells, embryos or wing discs [22]. In contrast, mutations of the palmitoylated cysteine to alanine (A) in several Wnt proteins, do not result in complete lack of activity [22], [23], [24], just as we observed for Wnt8aCA. Global expression of Wnt8aCA affected *Wnt8a* target genes during gastrulation and led to anterior head truncation at 24hpf. In contrast to Wnt8aCA, Wnt8aCY does not cause lack of anterior neural structures. While both mutations should affect the formation of the disulfide bond suggested by the XWnt8 crystal structure, replacing the cysteine with the more bulky tyrosine aminoacid (instead of the small alanine) might result in a structural conformation of Wnt8 protein which is not compatible with its function. Defects in secretion of Wnt-mutant proteins are usually associated with protein misfolding [44]. Our results show that Wnt8aCY still forms punctate structures that are targeted to the plasma membrane and filopodia of producing cells, indicating that the cysteine is not essential for these steps. This finding also suggests that lack of function of Wnt8aCY is not due to misfolding of the protein, consistent with results from cell culture experiments, showing that single or double mutants display a similar cell surface and intracellular distribution to WT Wnts [24]. The recent identification of Tiki as a matrix metalloproteinase that inactivates Xenopus Wnt8a by cleavage at its N-terminus, close to C55 might provide an alternative explanation [25], [45]. Since zebrafish and Xenopus Wnt8a proteins are highly conserved, the CY mutation in zebrafish might render Wnt8a more prone to cleavage by Tiki, thereby resulting in a more easily inactivated Wnt8a molecule.

A very surprising finding is the fact that, contrary to the single mutation Wnt8aCA or SA, which retain partial signaling activity, the double mutant Wnt8aCASA completely lacks signaling activity. In addition to impaired secretion, this mutation results in lower number of Wnt8a puncta not only in receiving tissue but also in Wnt producing cells which suggests that the two residues act synergistically to produce a fully active secreted Wnt protein. The complexity of Wnt biogenesis and signaling is illustrated by recent findings on the requirement of dedicated chaperones and modulators [46], [47], [48], [49], [50]. Understanding how the conserved residues affect the Wnt8 interaction with these molecules will provide new insights into the regulatory mechanisms of this pathway.

## Methods

### Animal care and handling

Fish were maintained at 28°C under standard conditions [51]. Embryos were staged according to morphological criteria [52] or in hours post-fertilization at 28°C. Wild-type strains used in this study were AB and TL.

### Whole mount *in situ* hybridization and antibody staining

Whole-mount mRNA *in situ* hybridization was carried out as previously described [53]. Digoxigenin-labeled probes were prepared from linearized templates using an RNA labeling and detection kit (Roche) as previously described [15]. Whole mount antibody staining was performed according to standard procedures. Antibodies used were: mouse monoclonal anti-β-catenin, clone 15B8 (Sigma, 1∶1000), rabbit polyclonal anti-GFP (Invitrogen, 1∶2000), goat anti-mouse Alexa546 and goat anti-rabbit Alexa488 (Invitrogen, 1∶500).

### DNA constructs

The C-terminal Wnt8a fusion proteins were generated by sub-cloning *Wnt8a* (ORF1) coding sequence into the pCS2+-Venus vector or pCS2+-GFP. Wnt8a protein alignment was done in JalView2.7 [54] using mafft with defaults. Mutant constructs were created by introducing point mutations in Wnt8a-Venus and Wnt8a-GFP with the Quick Change Site-Directed Mutagenesis Kit (Stratagene). The C-terminal Fzd9b fusion protein was generated by sub-cloning *fzd9b* coding sequence into the pCS2+-RFP vector [55]. Membrane-bound RFP contains a cDNA fragment of the C-terminal region (158–188 amino acids) of Xenopus K-ras and was previously described [56]. GFP-GPI [57] was cloned into pCS2+ and the GFP was exchanged with RFP to obtain the RFP-GPI construct.

### Luciferase Reporter Gene Assays

HEK 293T cells were transiently transfected with either untagged Wnt8a, WT or mutant Wnt8a-GFP plasmids together with TCF/Lef firefly luciferase reporter pGL3 BAR (pBAR, 20 ng) [28] and normalization control pGL4.73 hRLuc/SV40 (RLuc, 5 ng) (Promega, Madison) in triplicates. At 24 hours post transfection, firefly and renilla luciferase activities were measured using the Dual-Luciferase Reporter Assay System (Promega) and firefly activities were normalized to renilla luciferase levels as previously described [48].

### Secretion assay

HEK293T cells were transfected with 2 µg of either the WT or mutant Wnt8a constructs in 6-well plates. At 48 hours post transfection, conditioned media of cells were collected and concentrated using a centrifugal evaporator. Cells were lysed with passive lysis buffer (Promega). Lysates and media were subjected to anti-GFP Western blotting as described previously [49]. Band intensities were measured as outlined in http://lukemiller.org/index.php/2010/11/analyzing-gels-and-western-blots-with-image-j/.

### mRNA synthesis, injections and transplantations

Capped mRNA was synthesized using mMessage mMachine Kit (Ambion), following the manufacturer's protocol. RNA was diluted to 1 µg/µl in distilled, sterile H_2_O and stored at −80°C. The amount of mRNA injected was estimated from the concentration and volume of a sphere of RNA solution (with 0.2% Phenol Red) injected into oil at the same pressure settings −0,5; 1 or 1,8 nl of a 200 ng/µl solution. For global or mosaic expression, mRNA was injected in one blastomere of one-cell or 32 to 64-cell stage embryos, respectively. Transplantations were carried out at shield-stage using trimmed borosilicate capillaries. For detection of transplanted cells following *in situ* hybridization, donor embryos were injected at the one-cell stage with 0,05% biotin-coupled tetramethylrhodamine dextran (*M*r 10,000, Invitrogen, D3312). Transplanted cells were visualized by using the Vectastain ABC system (VectorLabs) and DAB (Sigma).

### Image acquisition and quantification

Confocal images were acquired using an LSM510-Meta confocal microscope with an Achroplan 40x/0.8 W dipping objective (Carl Zeiss Microimaging). For colocalization studies, GFP/Venus and RFP channels were acquired by sequential scanning. To acquire 3D time-lapse movies, 5-7 z-planes with a step size of 1−2 µm were recorded over a time interval of approximately 15 minutes. Z-stacks and 3D time-lapse movies were analyzed and quantified using the Zeiss Image Browser, Volocity (Improvision) and/or Fiji (http://fiji.sc/Fiji). To test statistical significance, p values were determined in Microsoft Excel (unpaired t test, two-tailed distribution). In all plots, error bars represent standard error of the mean (s.e.m.).

## Supporting Information

Movie S1 **Wnt8a is released from filopodia.** Confocal time-lapse movie on live embryos with mosaic coexpression of Wnt8a-Venus (green) and RFP-GPI (red). Two Wnt8a puncta are released from a filopodium of a Wnt8a producing cell (white square). A single confocal plane is shown; time is indicated in seconds (s).
(MOV)

Figure S1 **β-catenin protein has graded expression in the blastoderm margin.** Comparison of β-catenin staining and *wnt8a* ISH of shield stage embryos. (A) β-catenin staining intensity decreases from the margin towards the animal pole (left) but is not restricted to the *wnt8a* mRNA expression domain (right). (B) High magnification shows nuclear β-catenin staining at the margin and decreasing intensity from the margin towards the animal pole (left) but clearly detectable anteriorly (towards the animal pole) to the *wnt8a* mRNA expression domain (right). (C) β-catenin staining in the animal pole is restricted to the membrane. Dashed line indicates the blastoderm margin, * indicates a row of EVL cells. A and B are representative images of different embryos, at 90° from the shield. Scale bars: 50 µm.
(TIF)

Figure S2 **Time dependent β-catenin response to Wnt8a.** Single confocal sections at the animal pole of shield stage embryos stained with an antibody against β-catenin. (A–C) β-catenin staining is restricted to the membrane in a WT embryo. (D–I) Cells derived from embryos injected with a lineage tracer (red) and *wnt8a*-venus mRNA transplanted into WT host embryos. In embryos fixed 15 minutes after transplantation nuclear β-catenin is not detected around the transplanted cells (D–E). In embryos fixed 1 hour after transplantation strong nuclear β-catenin is detected around transplanted cells (G–I). Embryos injected with a lineage tracer (red) and *wnt8a*-venus RNA at the one-cell stage have no nuclear β-catenin (J-L). (A,D) Red channel, (B,E) green channel, (C,F) overlay. Scale bars: 50 µm.
(TIF)
