# Supplementary figures and images for: Dynamic Association with Donor Cell Filopodia and Lipid-Modification Are Essential Features of Wnt8a during Patterning of the Zebrafish Neuroectoderm
Source: PLoS One. 2014 Jan 10;9(1):e84922. doi: 10.1371/journal.pone.0084922 (PMC3888416; doi:10.1371/journal.pone.0084922)

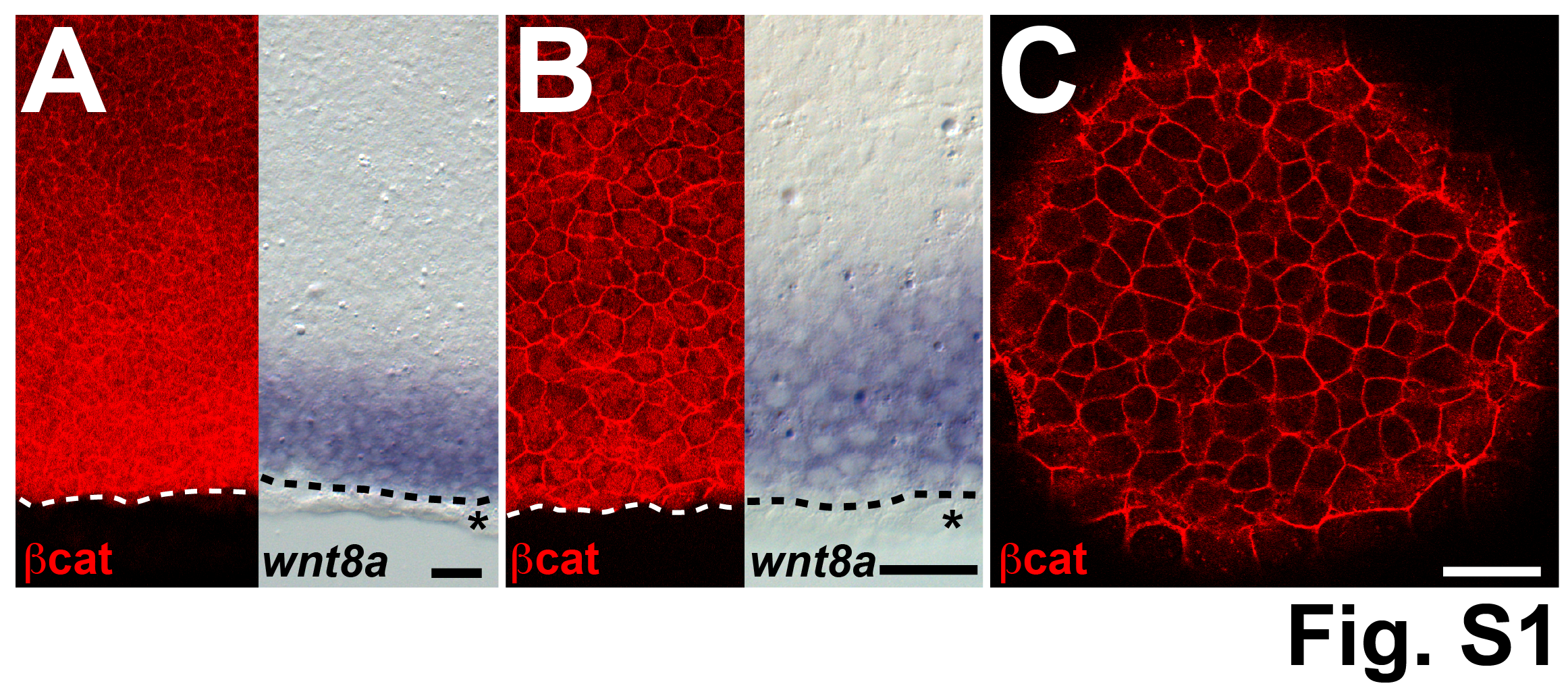

Supplement: Figure S1 — β-catenin protein has graded expression in the blastoderm margin. Comparison of β-catenin staining and wnt8a ISH of shield stage embryos. (A) β-catenin staining intensity decreases from the margin towards the animal pole (left) but is not restricted to the wnt8a mRNA expression domain (right). (B) High magnification shows nuclear β-catenin staining at the margin and decreasing intensity from the margin towards the animal pole (left) but clearly detectable anteriorly (towards the animal pole) to the wnt8a mRNA expression domain (right). (C) β-catenin staining in the animal pole is restricted to the membrane. Dashed line indicates the blastoderm margin, * indicates a row of EVL cells. A and B are representative images of different embryos, at 90° from the shield. Scale bars: 50 µm. (TIF) [file pone.0084922.s002.tif]

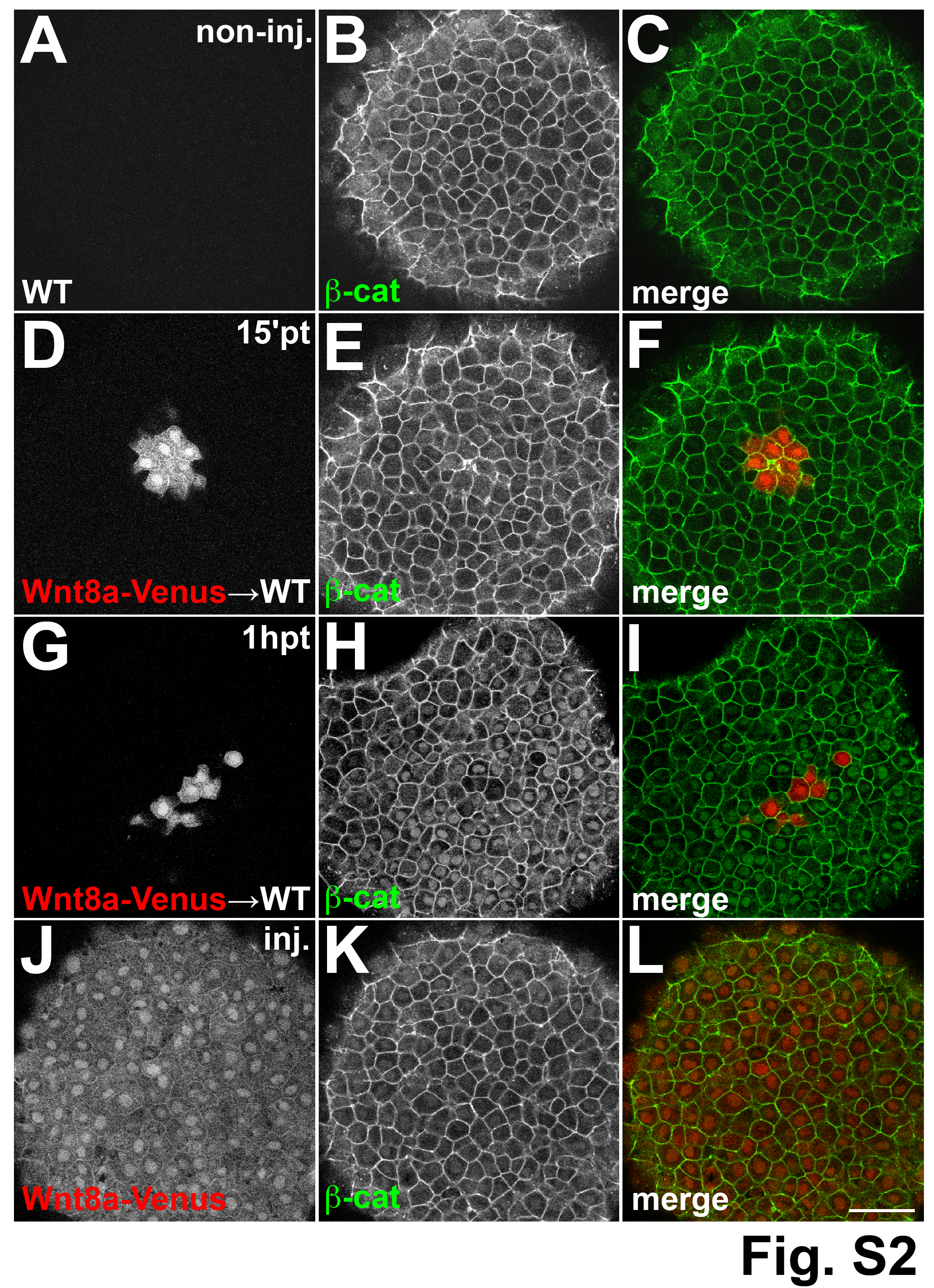

Supplement: Figure S2 — Time dependent β-catenin response to Wnt8a. Single confocal sections at the animal pole of shield stage embryos stained with an antibody against β-catenin. (A–C) β-catenin staining is restricted to the membrane in a WT embryo. (D–I) Cells derived from embryos injected with a lineage tracer (red) and wnt8a-venus mRNA transplanted into WT host embryos. In embryos fixed 15 minutes after transplantation nuclear β-catenin is not detected around the transplanted cells (D–E). In embryos fixed 1 hour after transplantation strong nuclear β-catenin is detected around transplanted cells (G–I). Embryos injected with a lineage tracer (red) and wnt8a-venus RNA at the one-cell stage have no nuclear β-catenin (J-L). (A,D) Red channel, (B,E) green channel, (C,F) overlay. Scale bars: 50 µm. (TIF) [file pone.0084922.s003.tif]
